# The impact of within-student and between-student variability in basic psychological need satisfaction on situational interest development

**DOI:** 10.3389/fpsyg.2025.1696225

**Published:** 2025-12-18

**Authors:** Alexander Minnaert

**Affiliations:** Department of Inclusive and Special Needs Education, University of Groningen, Groningen, Netherlands

**Keywords:** project-based education, cooperative learning, self-determination theory, motivation, education-to-work transition, VET

## Abstract

**Introduction:**

To prepare students for effective workplace-based learning and cooperation, it is necessary to have insight into students’ basic psychological need satisfaction and interest development over time.

**Methods:**

The framework of self-determination theory was used to conduct a field experiment, situated within a project-based cooperative learning setting, involving 169 students enrolled in higher secondary vocational education and training. These students were enrolled in a hands-on business administration and control specialist project, which required working in small learning groups. During this six-month project, students were repeatedly asked to complete the Quality of Working In Groups Instrument, an on-line measure of how strong the feeling-related need experiences of competence, autonomy, relatedness, and situational interest are fulfilled.

**Results:**

The unconditional means model showed that within-students’ variability in psychological needs and situational interest (varying from 51 till 84%) was huge over time. Furthermore, multi-level growth curve modelling showed that students’ growth in situational interest was positively predicted by students’ initial need fulfilment of autonomy and relatedness and by students’ growth in competence and autonomy satisfaction over time.

**Discussion:**

In line with self-determination theory, the findings underscore the importance of taking the basic psychological need satisfaction of students into account, tapping both between-students’ and within-students’ variability over time. To prepare vocational students for workplace-based learning, learning environments have to monitor, support, and fulfil students’ psychological needs and situational interest over time.

## 1 Introduction

A systematic review has well documented that differences in student engagement are related to perceived characteristics of the learning environment (Stroet et al., 2013). Researchers have also extensively documented that indicators of the quality of experiences in learning settings are closely related to the level of interest in the actual learning task (e.g., Hidi and Renninger, 2006; Debnath et al., 2007). Key questions remain regarding how situational interest develops in a particular area of study and to what extent (fluctuations in) this development is dynamically indexed by differences between students and by differences over time within students.

Astonishingly, this dynamic focus on situational interest is understudied within project-based, computer-supported cooperative learning settings (Minnaert et al., 2011; Hwang et al., 2015). In order to prepare students for engagement in cooperative project-based and workplace-oriented learning environments, emphasis is placed on students learning to share their process- and product-oriented experiences with their peers and coaches. Awareness and sensitivity to (changes in) the environment is the crux of the global Environmental Health and Safety (EHS) Policy. Although ample attention is devoted to tackling technical issues, little attention is spent on human drivers that address communication and processing capabilities with respect to EHS (Thibaud et al., 2018). Regarding social safety and occupational health, the OECD (2019) emphasizes the importance of fostering future employees’ transformative competencies: the ability to create new value, reflect, evaluate, regulate, and reconcile variability in one’s own needs, experiences, and goals across a variety of real-world settings to be prepared for an inclusive and sustainable world.

To optimally prepare students for a variety of learning environments, contextual characteristics affecting students’ developing interests are highly requested, though there is still a huge gap between education-based and workplace-based learning. Several authors (see, e.g., Schiefele, 2001; Schraw and Lehman, 2001; Hidi and Renninger, 2006; Soemer et al., 2019) have demonstrated the relationship between interest and various indicators of academic learning (e.g., achievement, learning from text, reading comprehension, and depth of learning). Others have emphasized the relationship between interest; autonomous motivation; and experiences of autonomy, competence, and relatedness (see, e.g., Alexander and Wade, 2000; Lewalter and Krapp, 2004; Mouratidis and Lens, 2015; Garn et al., 2018; Yu and Levesque-Bristol, 2020). A few have articulated the promising future of taking students’ (online, technology-enhanced) interest into account in both formal and informal learning stages of engagement and self-regulated effort (Hwang and Wu, 2014; Ryan and Deci, 2018; Sutarmina et al., 2025). In the overview of Järvelä and Renninger (2014), it was concluded that studies addressing interest, motivation, and engagement provide convergent evidence that design principles for learning need to account for differences of interest, motivation, and engagement across learners and across situations to trigger, sustain, and support the development of learners’ interest. In doing so, learning environments should meet the design features (1) supporting content-informed interactions and (2) providing scaffolding interactions for engagement, namely for learners to think and to work with the content.

Research has amply shown fluctuations in the development of motivation over time and across contexts (see Turner and Patrick, 2008; Järvelä and Renninger, 2014), indicating a general decline in motivation among adolescents to be associated with a decline in the satisfaction of the basic psychological need for competence, autonomy, and relatedness (Gnambs and Hanfstingl, 2016). By means of advanced structural equation modelling, Garn et al. (2018) showed that among Australian adolescents, the stability coefficients of autonomous motivation became small and non-significant, suggesting that longitudinal fluctuations in global levels of need satisfaction require far more attention across different time periods. Moreover, Fernet et al. (2020) found that within-person changes in self-determined work motivational profile membership occurred for 30 to 42% of health care nurses in 24 months. Rather surprisingly, most changes were encountered in the strongly motivated and in the self-determined motivated profiles, of which 25%, respectively, 28% moved to the Poorly or Moderately motivated profile. Hence, Fernet et al. (2020) emphasize the dynamic character of motivation, urging further studies to unfold the predictors of change in motivation not only among employees but also among upcoming employees to realize a smooth transition in education to work settings. Prior to Fernet et al. (2020), motivational profiles were aimed at among first-year undergraduate students across 2 months in the study of Gillet et al. (2017). Based on (only) two measurements, it was concluded that the most motivated profiles were more stable compared to the poor and controlled profiles. These findings among undergraduates are in huge contrast with the workplace-related results reported by Fernet et al. (2020). What do we learn from this incongruity? Contextual differences do matter, hence longitudinal fluctuations in both within- and between-person perspectives need to be thoroughly unravelled and examined in relation to (motivational) outcomes.

The majority of motivational research embedded within self-determination theory (SDT) has focused on inter-individual, between-person variability and on relationships between the basic psychological needs at one or two moments. Only a minority of studies focused explicitly on changes within students over time. Hence, taking into account within-person, intra-individual variability stems from a more recent interest among SDT researchers (see, e.g., Murayama et al., 2017). Noteworthy, longitudinal within-person variability in motivation and academic emotions has gained more attention in the last 15 years (Minnaert, 2023), and multilevel modelling accounting for nested data structures and even for contrasting groups of (special educational needs) students is more recently brought to the fore (Yu and Levesque-Bristol, 2020; Beck and Jackson, 2021; Loopers et al., 2024). These statistical and methodological improvements to take both within-person and between-person variability into account, embedded within a nested structure of time moments within students, are of utmost added value to study these intra- and interindividual differences in basic psychological need satisfaction and situational interest over time.

According to self-determination theory, the fulfilment of the basic psychological needs, i.e., the need for competence, autonomy, and relatedness, is pivotal in growth towards autonomous motivation, wellbeing, and development (Ryan and Deci, 2018). The basic psychological needs theory is one of the mini-theories of the overall self-determination theory and stresses that satisfying these needs fosters intrinsically motivated behaviour and promotes wellbeing, whereas thwarting them can lead to controlled motivation and decreased wellbeing. Hence, this framework emphasizes the need-supportive factors in the environment to continuously foster motivation, i.e., to meet the basic psychological needs of learners or (future) employees. If these needs are thwarted within a learning or work environment, a substantial decline in (autonomous) motivation is to be expected, shifting downwards to controlled motivation or even resulting in a-motivation or dropout. The dynamic interplay between persons’ needs and environmental support is pivotal in the waxing and waning of motivation over time and across situations. Next to differences between persons, targeted attention to within-person differences over time and contexts is highly at stake to pave the way towards (the design of as well as the professionalization in providing) more equitable support and more inclusive environments conducive to social, emotional, motivational, and (meta)cognitive growth and development.

However, taking between-person and within-person variability in feeling-related experiences of competence, autonomy, and relatedness into account and thereby making use of multilevel growth curve analyses to model changes in situational interest over time, as well as factors to explain these changes within project-based cooperative learning settings, bridging education-based to workplace-based learning, is missing from the literature. Hence, the aim of the current study was 2-fold, namely (1) to investigate the proportion of within- and between-student variability in the basic psychological need experiences of competence, autonomy, relatedness, and situational interest, and (2) to examine whether (changes in) the basic psychological need experiences are associated with (changes in) situational interest over time.

## 2 Methods

### 2.1 Research design and instrumentation

In the context of self-determined group work, it has already been demonstrated that students’ engagement in a cooperative small-group project largely taps into their underlying need satisfaction (Minnaert et al., 2007). Situational interest expressed in a group project strongly reflected the satisfaction of students’ underlying basic psychological needs. Hence, in line with Appleton et al. (2008), interest is regarded as an appropriate criterion variable for psychological needs. To grasp this information, researchers must register the students’ experiential states in the habitat of and during the ongoing project. This calls for a recording method that can capture induced motivational states (quasi) online. When motivational states are recorded shortly after the event(s) that caused them, it is less likely that students will have forgotten their specific feelings, which are indexed by the situation at hand. Accordingly, an electronic instrument was developed to assess students’ experiences of the quality of group learning: the Quality of Working in Groups Instrument (QWIGI) (see Minnaert et al., 2011).

A field study was conducted within the context of a 6-month-long small group project fostering cooperative and self-regulated learning among students in higher secondary vocational education. Students and teachers made use of QWIGI. Each week of the project period between October 2022 and March 2023, this electronic instrument required students to complete 10 bipolar self-report items with two opposing statements located at either end of a 7-point Likert scale. Students completed these items online halfway through or at the end of the project days. All together, these items assessed students’ basic psychological needs, namely their experiences of competence, autonomy, and social relatedness combined with their degree of responsibility for learning in groups, and situational interest in the group project. Over time, profile reliability was 0.70 for competence, 0.82 for autonomy, 0.89 for social relatedness, and 0.86 for situational interest, which is more than sufficient for further analysis. Lienert and Raatz (1994) mentioned a coefficient of 0.50 as the lower limit of sufficiency.

### 2.2 Participants

Participants were 169 students (comprising 93 male students) enrolled in Dutch secondary vocational education and training (VET). These students were between 15 and 19 years old at the time of data collection and studied the track as business administration and control specialists. Written informed consent for participation in this study was provided by the participants’ legal guardians or by the students themselves, in accordance with the local legislation and institutional requirements of the Ethical Committee Pedagogy and Educational Sciences.

All students were involved in a project-based educational innovation project on small-scale business concerns, fostering self-regulated learning in students and more process-oriented teaching and coaching in teachers. Students came from different socio-economic classes and from different social and cultural backgrounds, representative of the population for higher secondary vocational education in the Netherlands.

### 2.3 Data analysis

Multilevel growth curve modelling was used to analyse the nested data of 169 students in VET over 6 months. To investigate the within-student and between-student variability in the basic psychological need satisfaction of competence, autonomy, social relatedness, and situational interest, MLwiN with ML-estimations was used to perform unconditional means models. To examine the growth in the relationship between each basic psychological need and situational interest, unconditional linear growth models were run. Furthermore, conditional multilevel growth modelling of the effects of competence, autonomy, and relatedness on situational interest was performed, also taking the initial levels of the basic psychological needs and their changes from the initial levels over time.

## 3 Results

The unconditional means models, calculated by MLwiN, indicate that a large amount of within-student variability over time was encountered for competence (66%), autonomy (62%), and relatedness (84%), indicating that the zone of changeability within the environment is likely to affect within-student fluctuations in basic psychological needs. Accordingly, between-student variability was 34% for competence, 38% for autonomy, and 16% for relatedness. These results emphasize the relative dominance of moment-to-moment differences in students’ basic psychological need experiences compared to differences between students. Given the total variance in situational interest, 51% is attributed to within-student variability over time; hence, 49% is attributed to differences between students.

The unconditional linear growth models indicated that the mean linear growth is negative for relatedness, i.e., a significant general linear decrease in relatedness over time. The growth rates of situational interest, competence, and autonomy were not significantly different from zero over time (see fixed effects in Table 1). Significant initial differences between students at the start of the project were identified for all variables involved. Concerning the rate of growth for competence and autonomy, significant between-student differences were found (see random effect in Table 1).

**Table 1:** General trend of changes over time.

	Situational interest		Competence		Autonomy		Relatedness	
	Means model	Growth model	Means model	Growth model	Means model	Growth model	Means model	Growth model
*Fixed effects*								
Intercept	7.595	7.540	7.636	7.777	7.585	7.564	13.949	16.211
Time (growth)		0.031		−0.079		0.013		−0.124**
*Random effects*								
Intercept	2.112**	2.117**	1.081**	1.120**	1.376**	1.637**	3.282**	3.845**
Covariance		0.000		−0.126		−0.160		0.000
Time (growth)		0.000		0.149**		0.122**		0.000
−2*loglikelihood	2740.31	2739.78	2638.99	2615.39	2711.73	2702.85	3995.85	3870.17

**p < 0.01.

Conditional multilevel growth modelling indicated that perceived competence, autonomy, and relatedness were good predictors of students’ linear growth in situational interest. Overall positive linear growth for situational interest (0.101**) was encountered, taking competence, autonomy, and relatedness into account (see left part of Table 2). All basic psychological needs significantly contributed to the prediction of situational interest. When zooming in on the initial level of interest and into changes from the initial level over time, the initial level of relatedness and autonomy predicted interest growth significantly, while changes from the initial level in interest (i.e., change over time in interest) were best predicted by changes in competence and autonomy (see right part of Table 2). For interest to grow, relatedness matters from the onset of the project. This is equally valid with respect to perceived room for autonomy. During the project, changes in the need for autonomy and in the need for competence significantly affected the growth of interest. Given the total amount of variance in situational interest and taking all variables, initial levels, and changes over time into account (see random effects in Table 2), the proportion of within-student variability (56%) compared to between-student variability (44%) emphasizes the great importance of the within-person perspective.

**Table 2:** Conditional multilevel growth models of basic psychological needs on situational interest.

	Situational interest			Situational interest	
	Estimate	95% CI		Estimate	95% CI
*Fixed effects*			*Fixed effects*		
Intercept	2.253		Intercept	3.559	
Time (growth)	0.101**	(0.021–0.181)	Time (growth)	0.076*	(0.014–0.138)
Competence	0.339**	(0.259–0.429)	Competence T1	0.058	(−0.092–0.208)
Autonomy	0.263**	(0.187–0.339)	Autonomy T1	0.149**	(0.001–0.297)
Relatedness	0.041**	(0.013–0.069)	Relatedness T1	0.152**	(0.074–0.230)
			Competence ΔT	0.097**	(0.063–0.131)
			Autonomy ΔT	0.061**	(0.029–0.093)
			Relatedness ΔT	0.003	(−0.009–0.015)
*Random effects*			*Random effects*		
Between-student	1.427** (47%)		Between-student	1.413** (44%)	
Within-student	1.585** (53%)		Within-student	1.832** (56%)	
−2*loglikelihood	2516.78			2420.15	

CI, confidence interval; T1, initial level (time point 1); ΔT = changes from initial level. *p < 0.05, **p < 0.01.

## 4 Discussion

Addressing the first aim of this study, the proportion of within-student and between-student variability in the basic psychological need satisfaction of competence, autonomy, relatedness, and situational interest showed substantial percentages (51 up to 84%) attributed to within-student variability. The largest amount of within-student variability is to be found in the need satisfaction of relatedness, which is a rather innovative finding as compared to between-student variability. All in all, the amount of within-student variability indicates that interest fluctuates substantially within students over time in a project-based cooperative learning environment, requesting room and time for targeted person-oriented coaching and, if necessary, fine-grained intervention. This is an important take-home message for education-based and workplace-based designers aiming at cooperative learning goals embedded within interactive learning and workplace environments.

With respect to the second aim and to the cooperative and self-regulatory goals of this project-based learning environment, it is noteworthy that the mean linear growth over time (across all students) was negative for relatedness and zero for situational interest. Only in case the individual’s basic psychological needs of competence, autonomy, and relatedness were fulfilled during the 6-month-long project, an overall positive linear growth for situational interest (0.101**) was found. Hence, situational interest is growing over time in case the basic psychological needs of competence, autonomy, and relatedness are fulfilled, which is fully in line with self-determination theory.

These findings confirm the importance of both the within-person and between-person perspectives on the basic psychological needs for the growth of situational interest over time, but also the involvement required from the teachers, tutors, and/or coaches to explicitly monitor and support the need for relatedness of each of the group members within the project teams. In line with earlier findings (Minnaert et al., 2011), a cooperative mindset and atmosphere within each team should not be taken for granted in secondary vocational education, though of all basic needs, it contributes the most to situational interest at the initial stage of the project.

To monitor and support students’ basic psychological needs in innovative, project-based cooperative learning environments, recursive bootstrapping of targeted refinements and/or tailored actions is required to optimize implementation fidelity and outcome effectiveness (see Boekaerts and Minnaert, 2003). In the same vein, Sutarmina et al. (2025) underscored the necessity for tailored support strategies, particularly for male and younger students, to boost self-regulatory skills and to maintain situational interest in an online learning environment. In addition, Pylväs and Nokelainen (2025) also underscored the importance of basic psychological needs satisfaction to prevent burnout and dropout intentions among Finnish vocational students, and especially for female and older students or students with less work experience, as they seemed more prone to experiencing burnout exhaustion. Hence, gender, age, and prior work experiences should be taken into account as potentially moderating variables in the relationship between need satisfaction and students’ (dis)engagement.

Furthermore, beneficial for the growth of situational interest are the initial levels of relatedness and autonomy and the changes over time in the need satisfaction for autonomy and for competence. All needs matter, though in a timely and sequential manner. This rather innovative finding is of the utmost importance for teachers and coaches to prioritize the need-supportive strategies of providing structure, autonomy-support, and involvement in a group-centred and person-centered way. The satisfaction of the basic psychological needs for relatedness and autonomy requires full attention at the start of the project to stimulate students’ interest. Gradually, the need satisfaction for competence becomes more important to maintain students’ interest. These findings, embedded within the framework of self-determination theory, converge with recent results on triggering and maintaining engagement with learning among gifted and non-gifted students (Snikkers-Mommer et al., 2024). All children benefit from the fulfilment of their basic psychological needs. Additionally, all children, regardless of their IQ level, indicate that the provision of structure (e.g., clarity of expectations and rules, guidance to do the task better/quicker, offering clear feedback and feed-forward to improve their learning for mastery) is crucial to maintain engagement, even in the face of resistance. These findings also converge with the recent research findings of Esqueda Villegas et al. (2025) on the voices of autistic learners in mainstream secondary education in both Mexico and the Netherlands. All autistic students valued different types of structure provided by their teachers, which increased their confidence in completing a task and provided a sense of mastery experience. Besides, these students rely substantially on both teacher and peer interactions to gain clarity on tasks and to move forward. In line with self-determination theory (Ryan and Deci, 2018), growth in task engagement is universally determined by the use of need-supportive strategies and by students’ experience of basic psychological need satisfaction.

### 4.1 Strengths, limitations, and recommendations

The longitudinal design, comprising weekly measurements of students’ need satisfaction and situational interest over 6 months, was essential for capturing both within-student and between-student variability. The embeddedness of data collection within a VET habitat ensures the ecological validity of the data. The computer-supported and brief instrument being used to tap students’ need satisfaction and interest was experienced as very user-friendly and technologically up-to-date compared to paper-and-pencil questionnaires.

In this study, gender, age, or previous work experiences were not explicitly taken into account. As mentioned above, we highly recommend adding these potential moderators to retrieve even more fine-grained data and to model the data even more in-depth. In doing so, mixed-method designs, e.g., combining the extended quantitative data with (retrospective) interviews, video-stimulated recall methods, or with panel discussions, might contribute even more to the epistemological growth in understanding students’ variability and predictors of change over time to support transitions (Minnaert, 2023).

### 4.2 Implications for VET and conclusion

The evidence-informed significance of this study for monitoring situational interest is noticeable. Students’ self-assessment of the conditions for learning in terms of their feelings of autonomy, competence, and relatedness seems relevant to track and substantially explain changes in situational interest. In line with Murayama et al. (2017), Garn et al. (2018), Sutarmina et al. (2025), and Pylväs and Nokelainen (2025), a relative stability of motivation is no longer the default, neither in education nor in workplace settings. Longitudinal fluctuations in global levels of need satisfaction and situational interest, examined from both between-student and within-student perspectives, are the new default. This requires much more attention and monitoring in order to improve the practice and professionalization in VET.

In line with the aforementioned recommendations of Järvelä and Renninger (2014), the findings stress the need for scaffolding by teachers/coaches and the need for the professionalization of their content-related monitoring and role to guide learning and motivational development in project-based cooperative settings. Only in this way potentials for successful pre- and in-service professionalization aiming at interest growth can be unleashed: monitoring students’ basic psychological needs (dis)satisfaction and unfolding the waxing and waning of these dynamic processes to prevent disengagement and dropout in educational environments, in workplaces, and in education-to-work transitions (Heuving et al., 2025; OECD, 2025; Pylväs and Nokelainen, 2025).

## Data Availability

The raw data supporting the conclusions of this article will be made available by the authors, without undue reservation.
